# Angiogenesis in temporomandibular joint osteoarthritis: an update on mechanisms and research advances

**DOI:** 10.3389/fmed.2026.1866187

**Published:** 2026-10-06

**Authors:** Lingyan Lai, Huaiquan Liu, Haiyang Kou, Shili Yang, Xinyan Zhang, Yunlin Xu, Yu Sun, Bo Chen

**Affiliations:** Guizhou University of Traditional Chinese Medicine, Gui Yang, China

**Keywords:** angiogenesis, cartilage degradation, inflammation, subchondral bone, synovial, TMJOA

## Abstract

Temporomandibular joint osteoarthritis (TMJOA) is a chronic degenerative disorder characterized by condylar cartilage degeneration, abnormal subchondral bone remodeling, and synovial inflammation. Its pathogenesis is complex, and effective clinical interventions remain limited. In recent years, the critical role of angiogenesis in TMJOA pathogenesis has garnered increasing attention. During TMJOA progression, angiogenesis interacts dynamically with bone metabolism, playing a key role in the imbalance of subchondral bone remodeling, amplification of synovial inflammation, and acceleration of cartilage degradation. Understanding the specific mechanisms of angiogenesis in TMJOA is of great significance for developing novel therapeutic strategies. This article systematically reviews the core regulatory network of angiogenesis in the pathological mechanisms of TMJOA and prospects future research directions, aiming to provide a theoretical basis for mechanistic studies and clinical translation in TMJOA.

## 1 Introduction

Temporomandibular joint osteoarthritis (TMJOA) is a degenerative musculoskeletal disorder characterized clinically by a progressive pain syndrome, often accompanied by progressive functional impairments of the joint—such as limited mouth opening and masticatory weakness—and morphological abnormalities including condylar wear and disc displacement (1). It primarily affects structures of the temporomandibular joint (TMJ), such as the condyle, articular disc, and associated muscles and ligaments, and represents a common subtype of temporomandibular disorders (TMD).

Recent clinical observations indicate an epidemiological trend toward earlier onset, broader impact, and a wider age distribution among TMJOA patients. Epidemiological surveys report a significantly higher incidence in females than in males; the prevalence is approximately 14.56% in individuals under 30 years, 28%–32% in those aged 30–80 years, and as high as 70% in the elderly population (2–8). Concurrently, the prevalence of TMJOA is rising rapidly among young adults and even children, already exerting varying degrees of adverse effects on masticatory function, craniofacial development, and the balance of oral parafunctions such as respiration and swallowing (4, 7–9). Overall, the impact of TMJOA extends far beyond a single joint disorder. It impairs chewing and speech functions, affects growth, facial aesthetics, and quality of life, and is frequently accompanied by psychological issues such as anxiety and depression. Consequently, the prevention and management of TMJOA have become a major focus of research.

The pathological mechanisms of TMJOA are consistently associated with structural damage and metabolic abnormalities of the condyle. Its initiation and progression revolve around three core features: synovial inflammation, condylar cartilage degeneration, and abnormal remodeling of subchondral bone. These three features do not exist independently but are interconnected through molecular signaling (e.g., inflammatory cytokines, matrix-degrading enzymes) and structural damage, forming a progressive cascade that collectively drives disease development (10). In recent years, extensive studies have demonstrated that angiogenesis plays a role in TMJOA progression (11–13). Angiogenesis precedes bone formation, and the osseous vasculature not only supplies essential nutrients, growth factors, hormones, cytokines, and chemokines for bone formation but also removes metabolic waste products. Additionally, it serves as a bridge connecting bone with surrounding tissues. On the other hand, bone functions as an endocrine organ, secreting various cytokines and non-collagenous proteins such as sclerostin and osteocalcin, which can influence vascular function and structure. Moreover, the mechanical properties of bone also affect surrounding vasculature. Thus, the “Tripartite Regulation Theory” of bone metabolism (14). Highlights the codependency of vascular supply, bone formation, and bone resorption. In TMJOA, this triad becomes pathological. A hallmark of this shift is aberrant angiogenesis, wherein uncontrolled vascular growth into normally avascular territories (e.g., synovium, cartilage) acts as a conduit for disease propagation. These neo-vessels facilitate the influx of pro-inflammatory mediators and progenitor cells, promote aberrant subchondral bone turnover, enable enzymatic degradation of the cartilage matrix, compromise the integrity of the osteochondral junction, and potentiate nociceptive signaling (15). Given its central role, anti-angiogenic therapy is rationalized as a viable intervention for TMJOA (16, 17). A detailed mechanistic understanding is therefore imperative to guide targeted therapeutic development (Figure 1).

**Figure 1:**
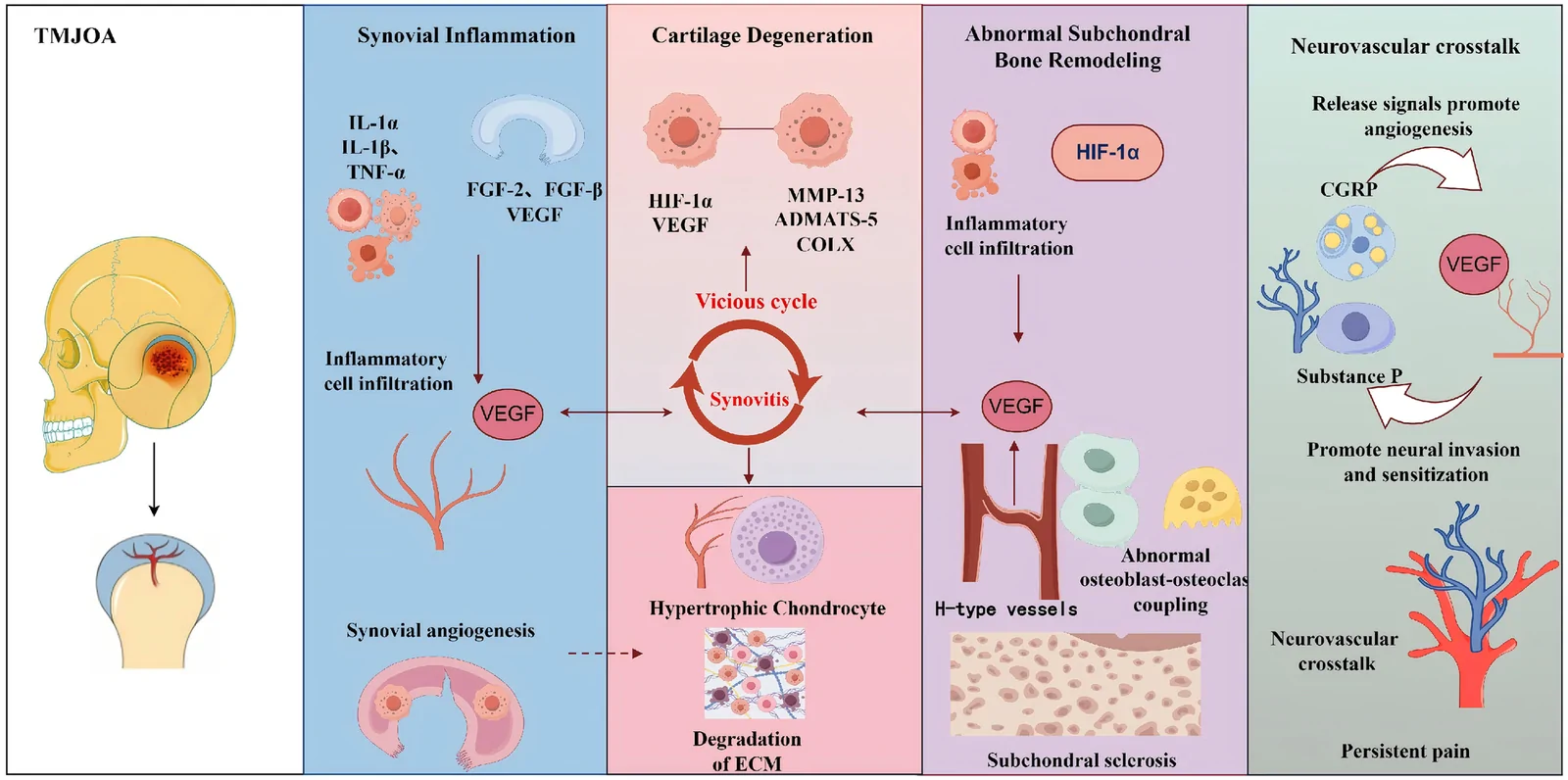
The pathological of TMJOA. All figures were created and exported using Figdraw 2.0 (https://www.figdraw.com), an online scientific illustration platform developed by Home for Researchers. According to Figdraw's copyright policy, all platform materials are original; images exported through the standard procedure are automatically licensed for publication with no risk of infringement. Accordingly, all figures included in this work are appropriately licensed. Figdraw is gratefully acknowledged for providing the illustration tools (project ID: POPRPf7355).

## 2 The mechanism and research progress of angiogenesis in TMJOA

### 2.1 The Impact of angiogenesis on synovial inflammation in TMJOA

Vascular alterations play a pivotal role in the pathological process of TMJ synovitis. Under inflammatory conditions, synovial vessels undergo dilation and congestion, accompanied by fibrin deposition and inflammatory cell infiltration. These vascular changes not only exacerbate synovial inflammation but may also contribute to increased intra-articular pressure and pain. These findings provide critical insights into the pathogenesis of temporomandibular joint disorders and lay the groundwork for developing therapeutic strategies targeting vascular-related pathological processes. Synovitis is characterized by inflammation of the synovial tissue, vascular dilation, and neovascularization, changes that are particularly prominent in internal derangements and osteoarthritis of the TMJ (18, 19). Histologically and arthroscopically, the vascular response in synovitis manifests as vasodilation, capillary congestion, and the formation of new blood vessels. Histological analysis has demonstrated a correlation between vascular proliferation in synovial biopsies and both joint space narrowing and synovial fold formation. Moderate to severe synovitis observed under arthroscopy—characterized by features such as capillary congestion and synovial hyperplasia—exhibits a high degree of concordance with histological diagnoses (18, 20). Studies utilizing animal models have elucidated the dynamic role of vascular responses in the initiation and progression of TMJ synovitis. In rat models, forced mouth opening or impairment of masticatory muscle function can induce synovial vascular dilation, fibrin deposition, and increased expression of inflammatory mediators (e.g., COX-2 and iNOS). These alterations are closely associated with angiogenesis and inflammatory cell infiltration (21, 22). Vascular Endothelial Growth Factor (VEGF) and Fibroblast Growth Factor-2 (FGF-2) are pivotal mediators promoting synovial angiogenesis in the TMJ. In patients with internal derangement of the TMJ, the expression of VEGF and its receptor Flt-1 within synovial tissues shows a significant positive correlation with microvessel density, indicating that the VEGF/Flt-1 signaling axis plays a predominant role in angiogenesis associated with synovitis (23).

Furthermore, angiogenesis exhibits a notable association with neural responses. Neuropeptides such as Substance *P* interact with vascular reactions in TMJ synovitis. Immunohistochemical studies demonstrate that Substance *P* is predominantly expressed in vascular endothelial cells of synovial tissue. Its expression levels are significantly higher in patients with internal derangement compared to controls, suggesting that Substance *P* may contribute to the pathological process of synovitis by modulating vascular permeability and promoting inflammatory cell aggregation (24). The release of pro-inflammatory factors like VEGF induces synovial tissue edema and sensitization of nerve endings, thereby contributing to pain generation. Experimental studies further confirm that VEGF levels in synovial fluid are significantly elevated before treatment and decrease following intra-articular hyaluronic acid injection, concomitant with improvements in clinical symptoms such as joint pain and mouth opening. This indicates VEGF's involvement in synovitis pathology and its potential as a therapeutic target (25). Although FGF-2 does not show significant changes, its synergistic interaction with VEGF may exacerbate vascular responses and inflammatory progression (23, 25). Mesenchymal stem cell (MSC) therapy has demonstrated efficacy in reducing synovial vascular dilation and fibrin deposition in osteoarthritis models, highlighting its anti-angiogenic and anti-inflammatory properties (26). Additionally, low-intensity pulsed ultrasound (LIPUS) therapy shows promise as a novel therapeutic direction for TMJOA by downregulating VEGF and IL-1β expression, thereby preventing growth impairment associated with synovitis (21).

### 2.2 Impact of angiogenesis on cartilage degeneration

Under physiological conditions, the articular cartilage of the TMJ maintains an avascular and aneural state. However, in the process of TMJOA, neovascularization breaches the “tidemark” from the subchondral bone and invades the cartilage tissue, initiating a destructive cascade. Angiogenesis and cartilage degeneration are closely linked through a pathological network involving mechanical stress sensing and inflammatory signal amplification. First, mechanical load influences angiogenesis and participates in the pathological progression of TMJOA by regulating the hypoxia-inducible factor-1*α* (HIF-1*α*)/VEGF signaling pathway (27, 28). VEGF-mediated angiogenesis is crucial for cartilage remodeling and ossification. VEGF is not only a potent pro-angiogenic factor but can also directly induce cartilage degradation. Studies have confirmed that VEGF expression is significantly elevated in osteoarthritic cartilage, and its level positively correlates with the degree of cartilage degeneration (29). In rats subjected to a hard-diet regimen, upregulation of VEGF and its downstream signaling molecules underscores the role of mechanical load in influencing cartilage angiogenesis and structural integrity via the VEGF pathway (30). Compressive stress induced by mechanical load creates a hypoxic state in cartilage. HIF-1*α* is a core factor through which chondrocytes respond to hypoxia. Under mechanical loading in the TMJ, HIF-1*α* helps maintain cartilage homeostasis by inducing VEGF expression. Studies show that in wild-type mice, mechanical stress leads to increased expression of HIF-1*α*, VEGF, and the apoptotic factor Caspase-3 in condylar cartilage, whereas HIF-1*α* heterozygous-deficient mice exhibit thinner cartilage and reduced proteoglycan content. This process mediates the release of cartilage-degrading enzymes such as MMP-13 and ADAMTS-5, which downregulate the expression of type II collagen and proteoglycans in the extracellular matrix, leading to matrix degradation and ultimately compromising the structural integrity of the cartilage (17, 31).

Second is the amplification of inflammatory responses. As an avascular tissue, disruption of cartilage homeostasis is often accompanied by abnormal angiogenesis and chronic inflammation, a phenomenon particularly evident in diseases such as osteoarthritis and rheumatoid arthritis. Recent research has revealed that the interplay between angiogenesis and inflammatory responses plays a pivotal role in TMJOA cartilage degradation: vascular endothelial cells promote aberrant vascular formation through pathways such as HIF-1, while immune cells like macrophages secrete pro-inflammatory cytokines, creating a vicious cycle (32, 33). In TMJOA cartilage, significant vascular invasion and ectopic ossification can be observed. Single-cell RNA sequencing has identified that M1-type macrophages enhance the migratory capacity of CD31⁺ endothelial cells by secreting pro-inflammatory factors (e.g., IL-6), while simultaneously stimulating chondrocytes to overexpress VEGFA. This tri-cellular interaction drives vascular infiltration into the avascular cartilage zone via the EGFR/ERK signaling pathway, establishing a “inflammation-angiogenesis” positive feedback loop (32, 33). In an inflammatory microenvironment, the IL-6/ERK/ERR*γ* pathway is activated, where ERR*γ*, as a downstream transcription factor, directly binds to the promoter regions of MMP9 and VEGFA, thereby coordinating extracellular matrix degradation and angiogenesis (33).

### 2.3 Impact of angiogenesis on subchondral bone remodeling

During the early stages of TMJOA, the loss of subchondral bone is considered a significant etiological factor in disease onset. Angiogenesis accelerates cartilage degeneration by promoting abnormal remodeling of the subchondral bone. Research indicates that angiogenesis within the subchondral bone not only alters the bone tissue microenvironment but also exacerbates cartilage destruction through the release of various pro-inflammatory and pro-angiogenic factors. For instance, the HIF-1 signaling pathway plays a key role in angiogenesis by regulating VEGF expression and promoting vascular infiltration into the subchondral bone. H-type blood vessels, predominantly exhibiting arcuate or columnar morphology, can be observed in the subchondral bone of the condyle (34). Osteoprogenitor cells are frequently clustered around these H-type vessels, coupling angiogenesis with osteogenesis in the condyle and thereby promoting subchondral bone remodeling (35, 36). The increase in H-type vessels may represent a central mechanism underlying cartilage degeneration and ossification. Notably, H-type vessel expression is elevated in the subchondral bone of osteoarthritic joints, where it contributes to abnormal bone remodeling and disrupts joint mechanical equilibrium (37). Furthermore, H-type vessels are recognized as a crucial link connecting subchondral bone remodeling with articular cartilage degradation. Their aberrant formation exacerbates both subchondral bone sclerosis and articular cartilage damage. Studies utilizing TMJOA animal models have confirmed a strong association between H-type vessels and TMJOA progression, demonstrating a significant increase in their density as the disease advances (37).

### 2.4 Impact of angiogenesis on pain

Angiogenesis plays a multifaceted role in TMJ pain, contributing not only indirectly by altering joint structure but also directly by participating in the transmission and modulation of pain signals. In TMJOA, angiogenesis is closely linked to processes such as inflammation, innervation, and bone remodeling, forming a complex pathological network. Targeting this network, particularly the process of angiogenesis, may offer novel therapeutic directions for managing pain in TMJOA. During the pathogenesis of TMJOA, vascular ingrowth is tightly coupled with neural outgrowth, both contributing to the development of OA-related pain. Neuropeptides such as calcitonin gene-related peptide (CGRP)and substance *P* act on corresponding vascular receptors to regulate blood flow and vascular permeability, while also promoting endothelial cell proliferation, migration, and angiogenesis (38). Sympathetic-derived neuropeptides, such as neuropeptide Y (NPY), act as acute vasoconstrictors and can significantly increase blood flow by promoting angiogenesis (39). Netrin −1facilitates vascular formation by regulating endothelial cell migration and inhibiting apoptosis (40). Studies have demonstrated a close synergistic relationship between angiogenesis and neuroinvasion in the progression of TMJOA. Single-cell RNA sequencing analysis has revealed that macrophages within the TMJOA condylar cartilage, particularly M1-like macrophages, concurrently promote both angiogenesis and innervation processes. Furthermore, chondrocytes in TMJOA exhibit high expression of genes associated with inflammation, angiogenesis, innervation, and ossification. These interconnected processes collectively contribute to the development of pain (32). Concomitant with vascular ingrowth, the formation of fragile and hyperpermeable pathological vascular channels allows inflammatory mediators (e.g., MMPs) and immune cells (such as macrophages) to infiltrate deeply. These factors may directly elicit pain by activating intra-articular nociceptors and altering neuronal excitability (41).

Angiogenesis, as a central regulatory node in the pathological progression of TMJOA, does not function in isolation but operates through a multidimensional network of “hypoxia–inflammation–neurovascular crosstalk,” profoundly contributing to disease onset and progression. In recent years, therapeutic research targeting angiogenesis in TMJOA has made significant breakthroughs, marked by a paradigm shift from “single-target vascular inhibition” toward “multi-target synergistic regulation.” Smart delivery systems such as electroresponsive hydrogels enable synchronized interventions, such as exRNA clearance coupled with VEGF suppression. Biological agents, including platelet-rich plasma and mesenchymal stem cells, promote cartilage repair by modulating angiogenic balance. Novel compounds such as HMGB1 inhibitors and artesunate have pioneered combined “anti-inflammatory and anti-angiogenic” therapeutic pathways. Collectively, these advances offer practical strategies for potential pathological reversal in TMJOA. Nevertheless, current research faces three major challenges:First, there remains a lack of precise identification of the dominant angiogenic regulators across distinct pathological stages of TMJOA—namely the early inflammatory phase, mid-stage cartilage degeneration, and late-stage osteophyte formation—which hampers the development of stage-adapted interventional strategies. Second, the molecular mechanisms underlying neurovascular crosstalk are not yet fully elucidated, and the vascular-neural signaling axis remains incompletely understood, limiting progress toward dual-target therapies that simultaneously alleviate pain and inhibit pathological angiogenesis. Third, bottlenecks persist in clinical translation. The intra-articular retention efficacy of smart drug carriers, as well as the diagnostic utility of angiogenesis-related biomarkers, require further validation through well-designed clinical studies (Table 1).

**Table 1:** Summary of relevant studies.

Ref. No.	Author & Year	Experimental model	Main detection indicators	Core research conclusions
(18–20)	Gynther et al., 1994; Sandler et al., 1998; Hakim et al., 2020	Human TMJ synovium from internal derangement/TMJOA patients	Arthroscopic synovial morphology, microvessel density, inflammatory cytokines IL-1*α*, TNF-α	Synovial angiogenesis and inflammatory infiltration are typical pathological markers of TMJ internal derangement.
(21)	Crossman et al., 2024	Juvenile rat TMJ arthritis model with LIPUS intervention	Synovial histology, VEGF, IL-1β expression	Low-intensity pulsed ultrasound inhibits VEGF and IL-1β to suppress pathological synovial angiogenesis.
(22)	Ozaki et al., 2008	Growing rat masseter weakness & jaw-opening synovitis model	Synovial vasodilation, fibrin deposition, COX-2, iNOS, VEGF	Masticatory muscle dysfunction induces angiogenic inflammatory mediators and synovial hyperemia.
(23)	Sato et al., 2003	Human synovium of TMJ internal derangement	FGF-2, VEGF, VEGFR expression, microvessel density	VEGF/FGF-2 signaling drives synovial pathological angiogenesis.
(24)	Sato et al., 2007	Human non-reducing disc displacement synovium	Substance *P* distribution, vascular permeability	Endothelial Substance *P* increases vascular permeability and recruits inflammatory cells.
(25)	Wang et al., 2021	Clinical patients receiving intra-articular hyaluronic acid	Synovial fluid VEGF, FGF-2, pain & mouth opening index	Synovial VEGF positively correlates with joint pain; hyaluronic acid reduces VEGF level.
(26)	Khazeni et al., 2024	Rat TMJOA model with MSC treatment	Synovial angiogenesis-related cytokines, synovial morphology	Bone marrow/adipose MSCs relieve synovial angiogenesis and inflammation.
(27)	Stojić et al., 2020	Rat mandibular lateral deviation model	Condyle/synovium/masseter VEGF, oxidative stress markers, nNOS, tissue histology	Mandibular lateral deviation disturbs VEGF, oxidative stress and NO signaling, triggering TMJ and muscle pathological remodeling.
(28)	Magara et al., 2012	Continuous compression rat disc cell model	Disc intermediate filament protein expression	Sustained mechanical compression alters matrix protein expression in TMJ disc cells.
(29)	Zhang et al., 2013	Rabbit experimental TMJ internal derangement model	Synovial VEGFR1, VEGFR2, VEGFR3 mRNA	Experimental TMJ internal derangement upregulates synovial VEGFR1/2 to promote joint adhesion.
(30, 31)	Jiang et al., 2017; Mino-Oka et al., 2017	Mechanical compression rat condylar cartilage	Cartilage proteome, HIF-1α, VEGF, MMP-13, ADAMTS-5, COLX	Hypoxia-activated HIF-1α/VEGF pathway elevates matrix-degrading enzymes to induce cartilage degradation.
(32)	Zhang et al., 2023	Human TMJOA condylar cartilage single-cell RNA-seq	Macrophage IL-6, endothelial VEGFA, chondrocyte matrix indicators	M1 macrophage-derived IL-6 forms positive feedback loop to accelerate angiogenesis and cartilage damage.
(33)	Zhao et al., 2019	Estrogen-related rat TMJOA model	ERR*γ*, VEGFA, MMP9 expression	ERR*γ* promotes angiogenesis and extracellular matrix degradation in TMJOA.
(34–37)	Lu et al., 2018; Ramasamy et al., 2014; Kusumbe et al., 2014; Li et al., 2021	Mouse mandibular condylar subchondral bone	H-type vessel density, osteoprogenitor distribution, osteoblast/osteoclast activity	Chondrocyte-derived signals boost H-type vessel formation; excess H-type vessels uncouple bone remodeling and worsen cartilage injury.
(38)	Leroux et al., 2020	DRG sensory neuron-endothelial co-culture	CGRP, Substance P, endothelial proliferation/migration rate	Sensory neuropeptides facilitate endothelial activation and regulate extracellular matrix remodeling.
(39)	Anderson et al., 2022	Skin tissue NPY functional verification model	NPY expression, endothelial angiogenesis activity	Neuropeptide Y mediates vasoregulation and pathological angiogenesis in inflammatory tissues.
(40)	Köhle et al., 2022	Fibromyalgia & osteoarthritis human serum	Serum Netrin-1 and its receptor concentration	Netrin-1 promotes endothelial migration and participates in degenerative joint angiogenesis.
(41)	Chung et al., 2023	Rodent TMJOA pain model	Vascular invasion, nerve hyperplasia, pain behavioral indexes	Pathological angiogenesis couples with nerve ingrowth to release inflammatory mediators and induce persistent TMJ pain.

## 3 Conclusions

Future research on angiogenesis in TMJOA should converge on two major directions: precision targeting and clinical translation. On the one hand, it is imperative to delineate the regulatory networks of angiogenesis across distinct disease stages through advanced technologies such as single-cell sequencing and spatial transcriptomics. This knowledge will underpin the development of stage-specific targeted therapies. On the other hand, efforts must be accelerated to translate intelligent delivery systems into clinical practice, and to identify reliable angiogenesis-related biomarkers in serum or synovial fluid that can facilitate early diagnosis. With deepening mechanistic insights into angiogenesis and continuous refinement of therapeutic technologies, intervention strategies based on vascular regulation hold the promise of shifting TMJOA treatment from symptom palliation toward pathological reversal, thereby opening new avenues for improving patient prognosis.
